# Extrapontine Myelinolysis following Extreme Hypernatremia and Hyperosmolarity

**DOI:** 10.1155/2019/7381597

**Published:** 2019-01-17

**Authors:** Jan-Niclas Schwade, Lior Haftel, Lars Rühe, Matthias Endmann

**Affiliations:** ^1^Klinik für Kinder-und Jugendmedizin, Evangelisches Krankenhaus Lippstadt, Wiedenbrückerstr. 33, 59555 Lippstadt, Germany; ^2^Radiologische Gemeinschaftspraxis am Evangelischen Krankenhaus Lippstadt, Wiedenbrückerstr. 33, 59555 Lippstadt, Germany

## Abstract

We present a case of a nearly 3-year-old girl who was admitted to hospital due to severe hypernatremia (196 mmol/l). Her medical history included central hypothyreosis and growth hormone deficiency. Rehydration and normalization of sodium was achieved according to guidelines. On the fourth day of hospitalization, the patient developed tremor, ataxia, and rigor. Cranial magnetic resonance imaging (cMRI) was performed and (mis)interpreted for meningoencephalitis, with corresponding diagnostic and therapeutic implications. The patient had extrapontine myelinolysis. The child recovered completely after hospitalization for nearly 2 weeks.

## 1. Introduction

Extreme hypernatremia is rare in infants, and rehydration therapy can be challenging, as sodium levels should be reduced very slowly [1]. There are various causes for hypernatremia including hypertonic dehydration or diabetes insipidus. Neurological complications of hypernatremia include brain edema, seizures, and coma. A rather rare complication can be extrapontine myelinolysis [2–4], which is often associated with an overcorrection of hyponatremia. Extrapontine myelinolysis (EPM) as well as central pontine myelinolysis (CPM) can occur due to changes in serum osmolality and are referred to as osmotic demyelination syndrome (ODS).

In this case report, we present the pitfalls of severe hypernatremia, its treatment and complications including (mis)interpretation of radiological imaging, and therapeutic conclusions.

## 2. Case Presentation

A 2-year-and-8-month-old Swiss girl presented to a pediatric office with fever of 38.8°C, vomiting, and refusal to eat for 3 days. Prior to admission, according to the parents, the patient had drunk ca. 500 ml of fluids. This was a response to a reviewers remark concerning signs of thirst. Blood analysis demonstrated severe hypernatremia (196 mmol/l), prompting urgent hospital admission.

The patient was born at term (40 + 1 weeks of gestation) and had a birth weight of 3390 g. She was delivered through a C-Section due to pathological cardiotocography (CTG) and green amniotic fluid. The APGAR score was 6/8/10. On the second day of life, she developed bilateral parenchymal and intraventricular grade III brain hemorrhage diagnosed by ultrasound. Additionally, she had recurring seizures, which were successfully treated with phenobarbital (3 mg/kg/d). cMRI at two weeks of age showed hydrocephalus with intraventricular hemorrhage in the caudothalamic groove displacing, but not including the thalamus, as well as a small intraparenchymal hemorrhage of the right parietal side and subarachnoid hemorrhage of the left occipital side, along the tentorium and the cisterna cerebellomedullaris, with signs for slight hypoxia.

Postnatally, the patient also had hypernatremia of 180 mmol/l, which was treated with infusion therapy (glucose 5%). We are not aware of any further urine or serum measurements (e.g. osmolality). Neonatal ultrasound showed, slight hyperplasia and no adrenal hemorrhage and tumor. She was discharged with a sodium level of 160 mmol/l. A “central dysregulation” etiology was hypothesized. Sodium levels in the first year were normal (or slightly elevated) and ranged from 136 to 154 mmol/l.

After birth, fT3 and fT4 serum levels were decreased; thus, thyroxine substitution was initiated. The therapy was ended after 1 month because of a hyperthyroid metabolic state (differential diagnosis at that time was euthyroid sick syndrome). Neonatal screening was unremarkable. Two weeks later, at the age of 6 weeks, fT4 was decreased again to 15.6 pmol/l (normal values 17–32), and thyroxine substitution was restarted.

At the age of 1 year, growth arrest (<3rd percentile, before 25–50%) as well as reduced oral intake were observed. IGF1 was reduced to 1.8 nmol/l (normal values 3.67–20.4), IGFBP3 was normal (1.07 mg/l), with no adrenal insufficiency (ACTH, cortisol, aldosterone, renin, FSH, LH, and prolactin normal), sodium was 136 mmol/l, no celiac disease, and bone age was normal. After re-evaluation of cMRI at 2 weeks of age, retrospective diagnosis of pituitary hypoplasia was carried out (ca 60 mm^3^, normal values 148 ± 37). Growth hormone deficiency was postulated followed by substitution with Norditropin. At the age of 12 months, cMRI was performed again, which showed partial pituitary dysgenesis and hydrocephalus malresorptivus.

The patient was regularly seen in endocrinological and neurological offices. She showed motor development delay which improved partially until the age of 2 years. At this age, a general development delay of 3–4 months and strabismus divergens/alternans on the left side were observed. She had one seizure at 18 months of age lasting 30–40 minutes with postictal paresis of the right arm and facial nerve paresis on the right side; cMRI at that time was unremarkable.

The patient was referred to our hospital in a reduced general condition. On presentation, the skin color was pale and turgor slightly reduced. She had symmetric limb movements with good muscle tonus but appeared tired. The pupils were equal and reacted promptly to light. The percentiles for weight (10.4 kg), length (93 cm), and head circumference (46 cm) were all below the 3rd percentile. Body temperature was 37.6°C, blood pressure was 97/65 mmHg, and heart rate was 102/min. The remainder of the physical examination was normal.

The patient was admitted to the pediatric intensive care unit. Blood gas analysis showed excessive hypernatremia (187 mmol/l) and hyperchloremia (148 mmol/l) with normal pH and base excess (Table 1). Osmolality was 362 mmol/kg (normal values 280–300 mmol/kg).

**Figure 1:**
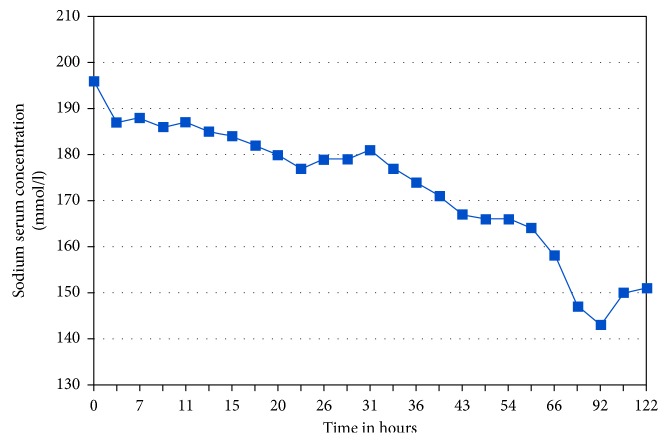
Sodium values over the course of hospitalization (in mmol/l, reference range 136–146 mmol/l).

Infusion with isotonic glucose-electrolyte solution (sodium 140 mmol/l and glucose 5%) was initiated. Potassium chloride (7.46%) was added due to mild hypokalemia of 3.02 mmol/l. Blood gas analysis was performed hourly, revealing a slow decrease of sodium to a minimum of 143 mmol/l (Figure 1), decreasing by an average of 0.5 mmol/l per hour.

Blood glucose on admission was 11.1 mmol/l and normalized with rehydration, suggesting the high blood glucose on admission was due to stress hyperglycemia. Supplementation of thyroid and growth hormones was continued. On the day of admission, urinary excretion was slightly reduced (ca. 2.4 ml/kg/h), and on the second day, the excretion increased (ca. 4 ml/kg/h). There was no fever or edema, vital parameters were stable, and other laboratory tests showed no abnormalities.

The following values were measured on admission: Urine osmolality was 876 mosmol/kg (normal values 50–1200 mosmol/kg), urine antidiuretic hormone (ADH) level was 23.70 ng/l (normal values 1.3–42.4 ng/l), plasma aldosterone level was 7.0 ng/dl (normal values < 9.0 ng/dl), and copeptin pro-arginine-vasopressin (AVP) was 4.4 pmol/l (normal values 1.70–11.25 pmol/l [5]) The cortisol level was slightly elevated to 32.72 *µ*g/dl (normal values 5–25 *µ*g/dl).

After normalization of serum electrolytes on day 4 of hospitalization, the child developed tremors, particularly while standing, and a general reduction of movement was observed. A slight bilateral rigor of both arms and ataxia were observed. The rest of the neurological evaluation was normal.

**Figure 2:**
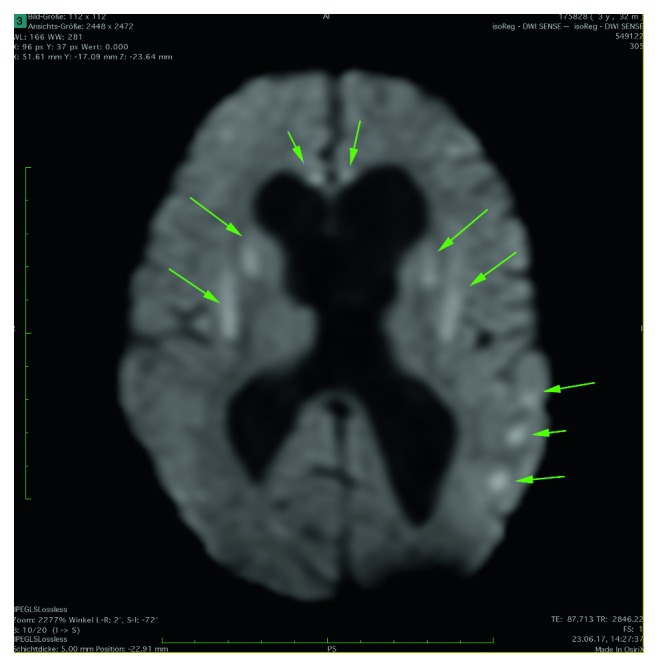
DWI—bilateral diffusion impairment in the basal ganglia, corpus callosum, and subcortical regions with slightly dilated lateral ventricles.

**Figure 3:**
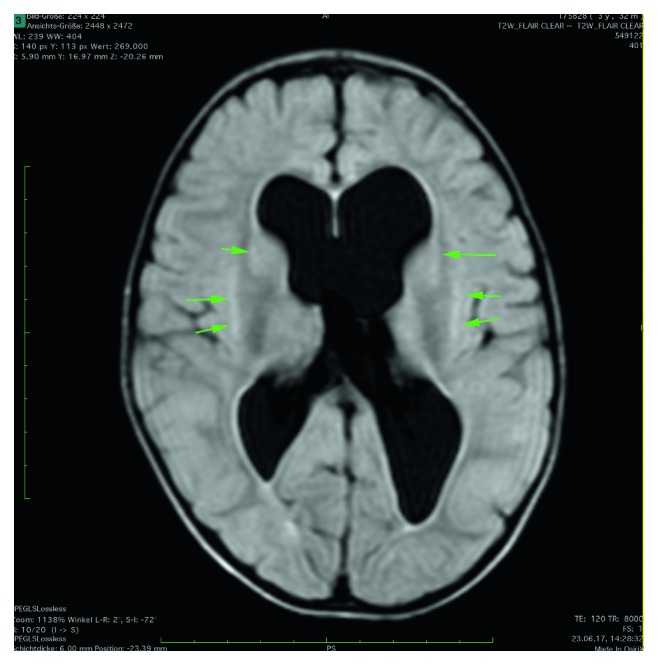
T2-FLAIR—discrete signal modulation in the corresponding T2-FLAIR sequence.

**Figure 4:**
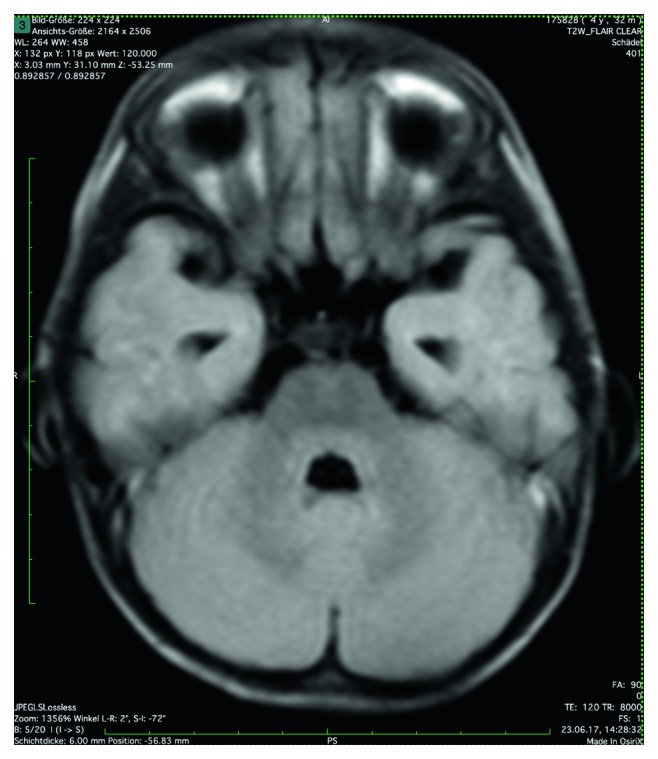
T2-FLAIR—unremarkable pons.

A 10/20-electroencephalography (EEG) was normal. MRI of the head showed vague, nonischemic diffusion impairment in the basal ganglia, corpus callosum, and subcortical regions on both sides (Figure 2), with only discrete signal modulation in the T2 fluid-attenuated inversion recovery (FLAIR) sequence (Figure 3). No signs for hemorrhage were observed. Slightly dilated lateral ventricles with no active hydrocephalus or indication of increase in pressure were observed. The pons was unremarkable (Figure 4). The findings were interpreted as meningoencephalitis, so lumbar puncture was performed.

The results of cell count, protein, and glucose in the cerebrospinal fluid (CSF) were normal. Cefotaxime and aciclovir intravenous were initiated. Multiplex polymerase chain reaction (PCR) of the CSF was negative for cytomegalovirus (CMV), *Cryptococcus neoformans*, *Escherichia coli*, Enterovirus, *Haemophilus influenzae* B, human herpesvirus 6 (HHV-6), human parechovirus, herpes simplex virus (HSV) 1/2, *Listeria monocytogenes*, *Neisseria meningitidis*, *Streptococcus agalactiae* (group B streptococcus (GBS)), *Streptococcus pneumoniae*, and varicella zoster virus (VZV). Additional PCR for Mycoplasma and Rotavirus was negative. The antibody specificity index (ASI) for mumps, measles, rubella, varicella, CMV, Epstein–Barr virus (EBV), herpes simplex and Borrelia, as well as tick-borne encephalitis (TBE) antibodies was normal. CSF examination showed an absence of oligoclonal bands, and only a slight increase of immunoglobulin M (IgM) of 2.4 mg/l (normal up to 1.3 mg/l) and a slight increase in albumin quotient of 8.5 (normal up to 4.2) were noted.

Creatine kinase (CK) was extremely elevated (12794 U/l), with normal values for other muscle enzymes (aspartate transaminase (AST), alanine transaminase (ALT)). Although CK-isoenzyme analysis was not performed, the elevated CK levels were assumed to be mostly brain creatine kinase (CK-BB). Rhabdomyolysis due to excessive hypernatremia remains a possible explanation. No seizures, as a possible explanation, were observed throughout the hospitalization.

After 5 days, the child was transferred by plane to Switzerland, where the family resides.

At the accepting hospital, the neurological symptoms remained. Rehydration was continued and slowly tapered over time. Brain computed tomography (CT) scan was performed to rule out sinus vein thrombosis. Antibacterial and antiviral therapy were stopped. Central dysregulation was discussed again concerning the origin of the hypernatremia. A retrospective review of the MRI was interpreted as extrapontine myelinolysis following extreme hypernatremia. After hospitalization for nearly 2 weeks, the child recovered completely.

## 3. Discussion

In emergency admissions, rapid evaluation of serum electrolytes and acid-base balance is indispensable. Imbalances require rapid medical intervention and application of evidence-based treatment using established guidelines when possible. Excessive hypernatremia is rare in infants and needs to be treated, as in this case, in a pediatric intensive care unit (PICU) due to high mortality.

In this case, hypernatremia could have been caused by hypertonic dehydration (vomiting and reduced oral intake), although the almost normal urine output of 2.4 ml/kg/h and normal urine osmolality are not typical of severe dehydration. Dehydration could have possibly been aggravated by a disturbance of thirst regulation (adipsic hypernatremia), which is often caused by intracranial lesions and injury to osmoreceptors [6, 7]. On the other hand, the patient had prior episodes of severe hypernatremia and might have been predisposed by the underlying disorder to extreme hypernatremia. This could be related to partial pituitary dysgenesis, central hypothyreosis, and growth hormone deficiency in our patient.

Rehydration therapy in hypernatremia is challenging for every pediatrician. Serum sodium levels should be carefully monitored and reduced very slowly (up to 0.5 mmol/l/h or 12 mmol/l/d) over time.

The child in our case report was treated according to established guidelines, and sodium levels were decreased slowly as recommended [1]. Still, the patient deteriorated on day 4, presumably due to extrapontine myelinolysis. Extrapontine myelinolysis as well as central pontine myelinolysis (CPM) mostly occur in osmolarity fluctuations and particularly when **hyponatremia** is overcorrected. There are some reported cases of children with extrapontine myelinolysis with **hypernatremia** [2–4], making myelinolysis an important differential diagnosis, especially if the patient deteriorates neurologically within a few days following the acute insult.

Patients with central pontine myelinolysis have a poor prognosis, with mortality of around 25%. Mortality has decreased in the last few decades, and even children with severe neurological deficiencies can have a complete recovery [8].

Although normalization of sodium levels was achieved according to guidelines, the development of subsequent neurological deficiencies on day 4 was puzzling and led to additional procedures. Impairments of diffusion in the basal ganglia, corpus callosum, and subcortical regions in the cMRI with normal angiography and only subtle signal variations in T2-FLAIR were misinterpreted as meningoencephalitis. The misinterpretation was probably because the radiologists not being aware of the extreme electrolyte imbalance on admission. The T2 “shine-through” effect can effectively be ruled out, as our radiologists intentionally use high B-values to prevent such an effect. Contrast agent administration was not performed.

Typical radiological features of ODS in the T2 sequence are hyperintensity in the central pons (CPM) or basal ganglia (EPM). Other possible locations include the hippocampus and lateral geniculate bodies.

Impairment is mostly symmetric, and in acute situations, a restricted diffusion (hyperintensity in DWI) with a lower apparent diffusion coefficient (ADC) is possible. These changes can often be reversible in subacute cases [9]. Enhancement after contrast agent administration is not common. In our case, the peripheral cortex was also affected, which is rare.

Patients should be assessed neurologically daily during rehydration therapy for pronounced electrolyte imbalance, aiming to detect neurological status changes promptly and to be able to respond quickly by modifying therapy regimes.

Imaging techniques should be performed in a timely manner, and interpretation requires integration of clinical context and laboratory findings to avoid misdiagnoses and treatment.

The following points can be learnt:

- Hypertonic dehydration can be life-threatening and often correlates with neurological complications (EPM and CPM).
- Quick and potentially repetitive radiological imaging is the diagnosis method of choice.
- Not every MRI enhancement means inflammation. Interpretation must involve active collaboration between radiologists and clinicians. Loss of information can lead to improper conclusions.

## Figures and Tables

**Table 1:** Initial blood gas analysis.

Parameter	Value	Unit	Reference range
pH	7.404		7.35–7.45
pO_2_	**70.8**	mmHg	83–108
pCo_2_	37.5	mmHg	32–45
SO_2_	95.6	%	95–99
CHCO_3_	23.4	mmol/l	20–26
ABE	−1	mmol/l	−3–2
Sodium	**187**	mmol/l	136–146
Potassium	**3.2**	mmol/l	3.5–5.0
Calcium	**1.30**	mmol/l	1.15–1.29
Chloride	**148**	mmol/l	98–106
Glucose	82	mg/dl	70–105
Lactate	1.1	mmol/l	0.5–1.6
Hb	**11.9**	g/dl	12.0–16.0
Co-Hb	0.8	%	0.5–1.5
Met-Hb	1.0	%	0.0–1.5
Bilirubin	0.4	mg/dl	0.2–1.2
